# Tendon Aging: A Silent Enemy Revealed Strategies for Effective Treatment

**DOI:** 10.1002/agm2.70027

**Published:** 2025-07-18

**Authors:** Wenhui Gu, Guohua Wang, Haifeng Zhang, Yinxian Yu

**Affiliations:** ^1^ Department of Physiology and Hypoxic Biomedicine, Institute of Special Environmental Medicine Nantong University Nantong China; ^2^ Department of Orthopaedic Surgery, Shanghai General Hospital Shanghai Jiao Tong University School of Medicine Shanghai China

**Keywords:** tendon, Tendon aging, Tendon Stem/Progenitor Cells

## Abstract

Tendons have a special makeup and set of physiological characteristics that make it difficult for them to mend themselves after damage. Aged tendons are more prone to injury, and wounded tendons will also age faster than usual. This creates a vicious cycle that might cause an injured tendon to reach surgery standards earlier than necessary or cause a re‐injury after surgery. Nowadays, the utilization of appropriate medications and exercise is the mainstay of tendon aging treatment. Due to their high potential for differentiation, tendon stem/progenitor cells (TSPCs) have drawn the attention of an increasing number of studies. The mechanism of tendon aging is outlined in this review, along with how it is caused by oxidative stress, hyperglycemia, inflammation, cell apoptosis, fatty infiltration, and its relationship to TSPCs. The goal of this review was to investigate the mechanism of aging and identify related treatment strategies that can effectively prevent further deterioration, slow down tendon aging, and minimize the risk of tendon re‐injury following surgery.

## Graphical abstract

This review commences with the mechanism of tendon aging, mainly introducing the aging of tendons induced by oxidative stress, hyperglycemia, inflammatory response, cell apoptosis, and fatty infiltration and the association with the tendon stem/progenitor cells, aiming to explore the mechanism of aging and find related treatment methods.

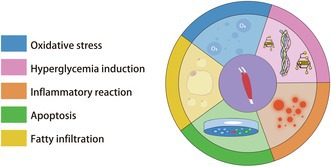

## 1 Composition and Pathological Features of Tendons

Tendons are mainly composed by tendinous tissue that constituted by tendon cells. Most of tendon cells are tendon fibroblasts, 90%–95% are tenoblast cells and others are tenocytes [1]. Tenoblast cells are activated in tendinous tissue upon tendon injury, resulting in the production of extracellular matrix that facilitates tendon recovery [2]. The extracellular matrix of the tendon will transmit the force [3] and primarily use mechanical deformation to alter the cellular actin cytoskeleton, thereby altering the shape, motility, and function of the cell [4]. Tendon cells adjust extracellular matrix renewal to physiological tissue load and pathological overload, the ability of tendon extracellular matrix that main composition is Type I procollagen thought to protect tendon homeostasis and health, When its homeostasis is deranged it is considered a risk factor for tendon‐related lesions [5]. And tendinous tissue are dense connective tissue, which also rich in Type I collagen [6]. Collagen helps maintain the integrity and diverse properties of tendinous tissue by forming collagen fibers and supporting structures. At the tendon junction, Type II muscle fibers have a smaller cross‐sectional area compared with Type I muscle fibers, rendering them more susceptible to strain [7]. The function of the tendon junction is to transfer force from the muscle to the bone [8] and typically lose less energy [4] or produce less deformation under greater loads, which is a collagen‐rich protrusion from the tendon that is formed by the muscle membrane being densely folded [7]. While mechanical strain can improve the diameter and collagenous fiber composition of a tendon, persistent mechanical tension strengthens the tendon's hardness [1]. If give the tendon repeated mechanical loads within the physiological tolerance can also damage the microstructure of the tendon, but these damages could be repaired by tenoblast cells [3]. Tendon strength is important for evaluating the mechanical properties of tendons; dynamic stretching increases tendon strength more than static stretching does [9] but, with the exception of overload training, strength training, whether short‐ or long‐term, can improve tendon strength and strain tolerance [10]. Exercise that is high‐strain isotonic could enhance the mechanical characteristics of tendons; nevertheless, the effects of various exercise regimens on tendon structures vary [11].

Tendon aging is an aging‐related phenomena that is defined by a degenerative process [12]—a sequence of aging‐related structural and functional changes in tendon tissue [1]. The function of peripheral blood arteries and tendon sheath cells declines with age, the blood supplement for tendon deteriorates, and adipocyte accumulation rises [13]. The structure of aged tendon cells changes, resulting in a loss in collagen fiber tissue, water volume, and proteoglycan, which leads to looser tissue [14]. As a result, the elasticity of the aged tendon declines and it is more susceptible to calcification [1]. Tendons that have been wounded [15] or aged [16] will generate more Type III collagen, which influences the tensile resistance of the tendon. However, Jessica's research [17] revealed that while aging did not significantly change the mechanical characteristics of intact tendons, it did lead to a reduction in the ability of tendons to heal. This was demonstrated by a decrease in maximum load, stiffness, and yield load as well as less collagen matrix bridging during aged tendon repair. Apoptosis or mechanical damage will produce a decrease in the quantity of tendon cells [4]. Age‐related changes in tendon nuclei's form and quantity may be due to a decline in tendon cells' capacity for repair and metabolic activity [18]. Elder's tendon's mechanical characteristics can be impacted by short‐term de‐loading, which can cause a decrease in collagen and compromise collagen turnover. This is why appropriate mechanical loading is crucial for tendon health [19]. But short‐term resistance training cannot improve the mechanical properties of tendon deteriorated with age, the function of the tendon worse may result by less activity of the elder [20]. In a study by Chen [21], TSPCs were found to accumulate in aged tendinous tissue. Matrix metalloproteinase (MMP) degrades protein components in the extracellular matrix, playing a significant role in tissue remodeling. Aged and injured tendons exhibit increased levels or overexpression of MMP [22]. In contrast to normal tendons, aging tendons experience tissue changes and aberrant expression patterns. These changes lead to pathological factors that accelerate tendon aging and impact tendon healing.

## 2 Role of Tendon Stem/Progenitor Cells in Tendon Aging

The stem cells divided from tendon called the stem/progenitor cells x(TSPCs) [23], in recent year more and more studies put their attention into the tendon stem or progenitor cells, a newly discovered less abundant cells in tendon [24]. TSPCs are found have the most important function to repair the injured tendon [21, 25]. When a tendon sustains an acute injury, TSPCs are activated to repair the damage; however, because there are less TSPCs in the tendon, the healing process may take longer [8]. Young TSPCs were smaller in size and spindle‐shaped, but aged TSPCs exhibited a star‐like flattened cell appearance [26]. Cell cycle could reflect the proliferation of the cells, in Rui's study found that the accumulation of the TSPCs were blocked in the G1/S phase [23]. In newborns, tendon have great ability of proliferation and differentiation could promote the injury healing better, but with aging, the ability of proliferation, renewal and cloning will decline [27]. The ability of proliferation decline shows in aged tendinous tissue with TSPCs aging [28]. Aged TSPCs will accompany with the decline of ability of migratory [26], which cause age‐related tendon diseases and influence the tendon's ability to repair and replace [29, 30]. Moreover, aged tendons themselves become harder due to calcification [1]. According to study by Kohler [26], aged or functionally deteriorating tendon except that it decreases the function of the cell, which would cause the tissue to deteriorate more quickly than young, healthy tissue. Thus, focusing on aging TSPCs offers new avenues for the treatment of tendon aging in the future.

## 3 Mechanisms of Aging in Tendons

**FIGURE 1:**
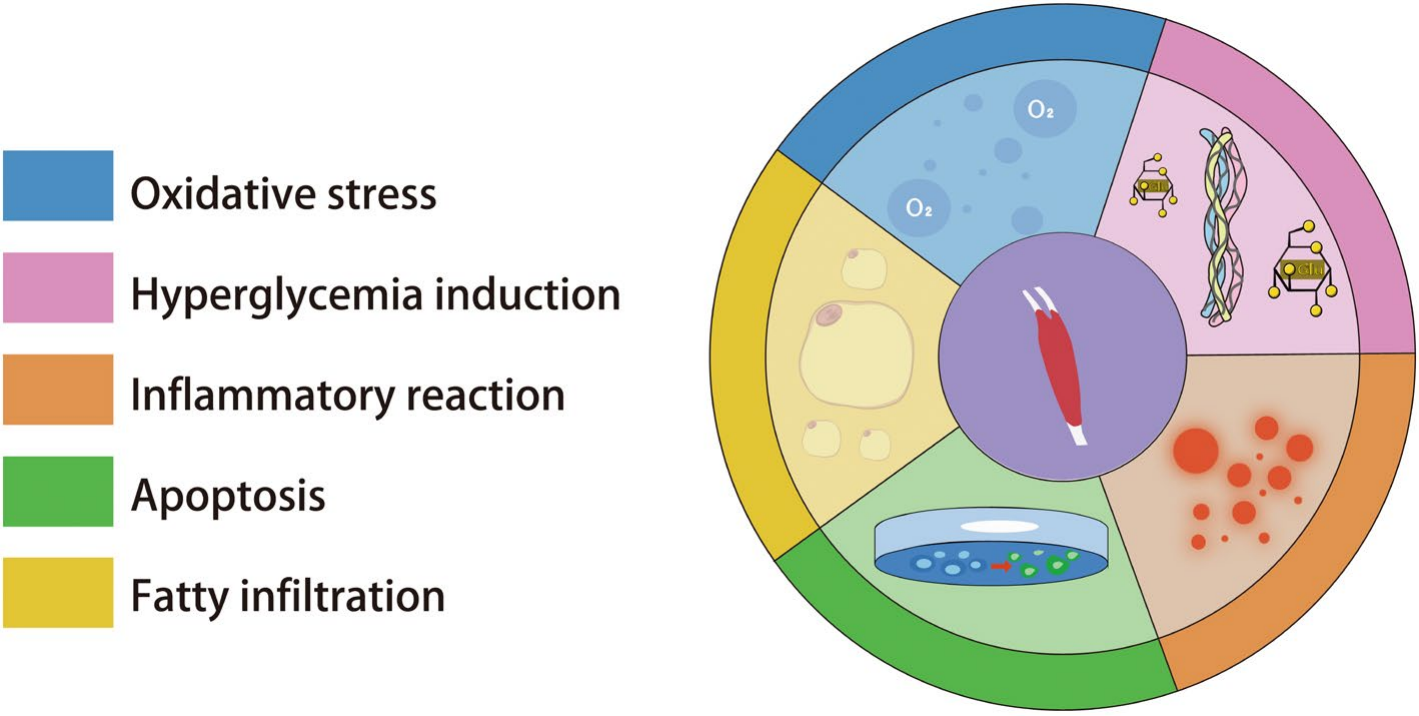
The mechanism of tendon aging.

Tendon aging is exacerbated by injury, as aged tendons are inherently more susceptible to damage. This process is influenced by oxidative stress, resulting from an imbalance in the body's antioxidant system [31], as well as hyperglycemia‐induced protein glycosylation, which exacerbates tendon fibrosis and diminishes elasticity [32]. Other contributing factors include chronic inflammation [33], excessive apoptosis [34], and fatty infiltration, all of which impair the regenerative capacity of tendon cells and hinder the healing process associated with tendon aging. TSPCs, which play a crucial role in tendon repair, are characterized by their ability to differentiate, setting them apart from mature tendon cells. However, under conditions that promote aging, TSPCs may undergo aberrant differentiation, giving rise to osteoblasts, chondrocytes, or adipocytes, further accelerating the process of tendon aging (Figure 1) [24]. While several mechanisms underlying tendon aging have been identified, it is important to note that tendon aging does not necessarily occur solely due to these factors, because healthy tendons may encounter these conditions during the repair process.

### 3.1 Oxidative Stress

**FIGURE 2:**
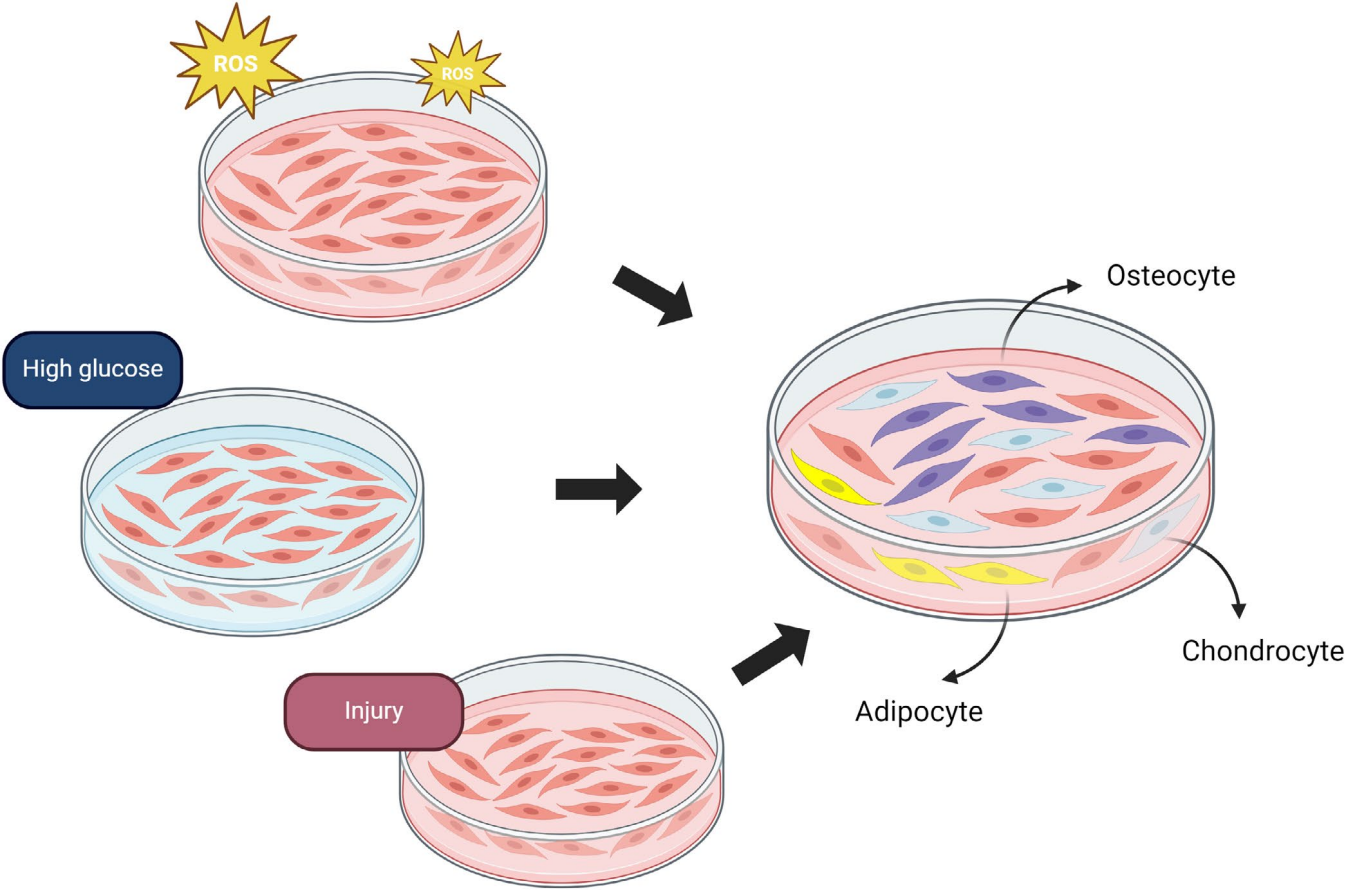
Describe aged TSPCs enhance the ability of abnormal differentiation.

When the cellular redox balance is disrupted, excessive production of reactive oxygen species (ROS) occurs. This phenomenon is exacerbated by the age‐related decline in the efficiency of the antioxidant system, which becomes insufficient to neutralize excess ROS, leading to oxidative stress and subsequent tissue damage [31]. Free radicals, characterized by unpaired electrons, react with surrounding molecules, including oxygen, generating peroxides that damage proteins and macromolecules like DNA, ultimately causing cellular injury. For example, hydrogen peroxide (H_2_O_2_) can generate free radicals, induce oxidative stress, and impair tendon cells, adversely affecting healing. Liu [35] reported that Alda‐1, through activation of mitochondrial aldehyde dehydrogenase 2 (ALDH2), reduces oxidative and endoplasmic reticulum stress, thereby preventing Achilles tendinopathy. Although hydrogen peroxide levels were shown to induce oxidative damage, activating endoplasmic reticulum stress and contributing to Achilles tendinopathy, Fu [31] found that low doses of hydrogen peroxide (50 μM) positively influenced tendon healing, whereas higher doses (500 μM) exacerbated damage. This dose‐dependent effect may relate to oxidative enzyme activity and vascularization, with higher concentrations promoting vascular responses that mitigate negative effects on tendons. Similarly, Yoshida [36] suggested that oxidative imbalance accelerates degeneration in rotator cuff tears. Nitric oxide (NO), a specific oxygen free radical synthesized by nitric oxide synthase (NOS), significantly increases in injured rat and human rotator cuff tendons compared to healthy tendons [37]. While a balance between ROS and NO supports physiological activities and tendon repair, excessive levels of either cause oxidative stress, damaging tendon tissue and accelerating aging. As a specialized subset of tendon cells, tendon stem/progenitor cells (TSPCs) face oxidative stress while maintaining their capacity for proliferation and repair. Research by Li [38] demonstrated that oxidative stress frequently occurs at tendon injury sites involving TSPCs and correlates with aging, jeopardizing their physiological and therapeutic efficacy. Oxidative stress in TSPCs promotes heterotopic ossification and is associated with increased osteogenic differentiation [39]. Reducing ROS levels, however, may mitigate heterotopic ossification and restore TSPC functionality [40]. Emerging treatments target ROS reduction to protect TSPCs. For instance, exosomes from human exfoliated deciduous teeth, containing bio‐nanoparticles, alleviate ROS in TSPCs, upregulate antioxidant genes, such as Sod1, Cat, Gpx1, and adjust the methylation of histones such as H3K9me3 and H3K27me3, inhibiting NF‐κB signaling and reducing tendon degeneration [41]. Additionally, Xue [42] reported that enhancing NAD+ metabolism increases circulating glutamine levels, which alongside other antioxidants, alleviates aging by improving oxidative stress. Mitochondrial dysfunction, largely driven by oxidative stress, is a key contributor to aging. Antioxidant supplementation, such as glutathione, has shown to shield mitochondria from oxidative damage, enhancing muscle strength, exercise capacity, and potentially delaying tendon aging [43]. Amiri [44] highlighted that resistance training combined with creatine monohydrate supplementation enhances glutathione peroxidase activity, bolstering the body's antioxidant system. This may reduce oxidative stress, preserve mitochondrial function, and improve overall motor performance, potentially delaying tendon aging. Moreover, the Nrf2‐HO1 signaling pathway plays a central role in antioxidative stress, with Nrf2 regulating redox homeostasis by modulating oxidase HO‐1 expression. Bone marrow mesenchymal stem cell‐derived extracellular vesicles treated with the antioxidant eugenol activate the Nrf2/HO‐1 pathway, enhancing TSPC resistance to oxidative stress and promoting healing [38]. Lower doses of vitamin C further support TSPC migration, viability, and proliferation, presenting another viable strategy for tendon repair [45]. Oxidative stress represents a critical therapeutic target in the treatment of tendon aging. By reducing ROS levels and regulating antioxidant pathways, it is possible to preserve the functionality of TSPCs, mitigate ectopic ossification and fibrosis, and thereby enhance tendon repair. Various antioxidants, such as glutathione and exosomal treatments [46], along with controlled doses of ROS, can promote healing, although maintaining appropriate concentrations is essential to avoid tissue damage. Combining antioxidant therapy with exercise interventions or other treatments can further amplify therapeutic outcomes, offering an effective strategy to delay tendon aging. These approaches highlight their therapeutic potential for addressing degenerative tendinopathies and age‐related TSPC dysfunction. The increase in ROS is not solely attributed to the imbalance of the body's antioxidant system but is also accelerated under hyperglycemic conditions. Hyperglycemia promotes ROS production through multiple mechanisms, including mitochondrial overload, activation of the AGEs‐RAGE signaling pathway, and depletion of antioxidant capacity via the polyol pathway. Therefore, it is essential to consider the role of other contributing factors leading to elevated ROS levels, which, in turn, accelerate tendon aging. The increase in ROS is not solely attributed to the imbalance of the body's antioxidant system but is also accelerated under hyperglycemic conditions. Hyperglycemia promotes ROS production through multiple mechanisms, including mitochondrial overload, activation of the AGEs‐RAGE signaling pathway [47], and depletion of antioxidant capacity via the polyol pathway [48]. Therefore, it is essential to consider the role of other contributing factors leading to elevated ROS levels, which, in turn, accelerate tendon aging (Figure 2).

### 3.2 Hyperglycemia Induction

Protein glycosylation and polysaccharide deposition increase with aging in tendons, leading to tendon fibrosis. The interaction between nonenzymatic glycation and oxidative reactions promotes the formation of early glycation products, which aggregate within collagen [49] and eventually form advanced glycation end‐products (AGEs), derived in part from albumin [50]. AGEs are generated through nonenzymatic reactions and are resistant to enzymatic degradation or catabolism. This process is exacerbated in individuals with Type 2 diabetes, characterized by chronic hyperglycemia, where elevated AGE levels are detectable in the plasma and serve as biomarkers for aging in diabetic patients, as shown in studies by Alexis [50] and Daniela [51]. In diabetes, impaired glucose metabolism contributes to excessive AGE accumulation, restricting the capacity of tendon‐derived fibroblasts to maintain extracellular matrix biosynthesis and homeostasis [52]. Furthermore, AGEs significantly disrupt the transcriptome of tendon fibroblasts [53], exacerbating the development of tendinopathy. AGE accumulation is also associated with aging‐related connective tissue damage [54], including tendon brittleness or stiffness [55], reduced collagen fiber sliding, delayed tendon healing [56], and impaired mechanical adaptability. As AGEs accumulate within collagen fibrils, they stiffen at high‐strain levels [57], diminishing the fibers' ability to elongate and relax under stress [54]. This impairs the tendons' ability to tolerate mechanical loads and varying exercise intensities. Elevated glucose levels further accelerate collagen and protein glycosylation, disproportionately affecting patients with Type 2 diabetes, even though AGE accumulation is a normal part of aging [49]. A study by Cronin [32] demonstrated that the Achilles tendon of long‐term diabetic patients exhibited greater susceptibility to damage and delayed healing, accelerating tendon aging. This is attributed to diabetes‐induced alterations in tendon tissue composition, resulting in reduced elasticity, diminished energy storage capacity, and impaired walking efficiency. Additionally, proteoglycan deposition at the tendon‐bone interface in the rotator cuff was reported to be higher in aged mice compared to young and adult mice in comparable animal models [14]. AGE accumulation, a hallmark of connective tissue aging, impairs tissue renewal [49], as evidenced in Tobias's study [58], which identified methylglyoxal as a key 1,2‐dicarbonyl compound driving nonenzymatic protein modifications and aging through Maillard‐derived processes. Oxidative stress is another contributor to AGE accumulation. Dehydroepiandrosterone, a multifunctional steroid with antioxidant properties, has been shown to reduce plasma glutaraldehyde levels and inhibit AGE formation in diabetic patients [59]. Conversely, high AGE levels exacerbate oxidative stress. Benfotiamine, a fat‐soluble vitamin B1, has been shown to mitigate oxidative stress by reducing endogenous AGE accumulation [59]. Aging and hyperglycemia are significant risk factors for AGE buildup, particularly in older diabetic patients, which accelerates tendon degeneration and impairs their regenerative capacity. Moreover, AGEs promote the aging of TSPCs, reducing their regenerative potential [60]. In a hyperglycemic environment, TSPCs undergo aberrant differentiation due to elevated HMGB1 secretion, which regulates this process via the RAGE/β‐catenin signaling pathway [61]. Additionally, macrophage migration inhibitory factor (MIF), an important regulator in diabetes pathogenesis, enhances the osteochondrogenic differentiation of TSPCs while inhibiting their tendonogenic differentiation, as noted by Kim [62]. MIF is thus a potential therapeutic target for diabetes‐induced tendinopathy. Collectively, these findings suggest that hyperglycemia not only accelerates but also mimics the aging process of TSPCs, ultimately contributing to tendon degeneration. Improving hyperglycemia can alleviate the accumulation of AGEs and prevent tendon fibroblasts from losing their ability to synthesize extracellular matrix due to abnormal glucose metabolism. Additionally, hyperglycemia accelerates the aging of TSPCs and oxidative stress. Both oxidative stress and AGEs can activate inflammatory pathways, such as NF‐κB and MAPK pathways, promoting the release of proinflammatory factors, which further exacerbate tendon fibrosis. Therefore, regulating hyperglycemia is crucial. Strict blood glucose control in daily life, particularly for diabetic patients through medication adherence, or by supplementing antioxidants such as vitamin C to inhibit the formation of AGEs, along with increased physical activity to mitigate tendon aging induced by hyperglycemia, can accelerate the repair of tendon injuries.

### 3.3 Inflammatory Reaction

**FIGURE 3:**
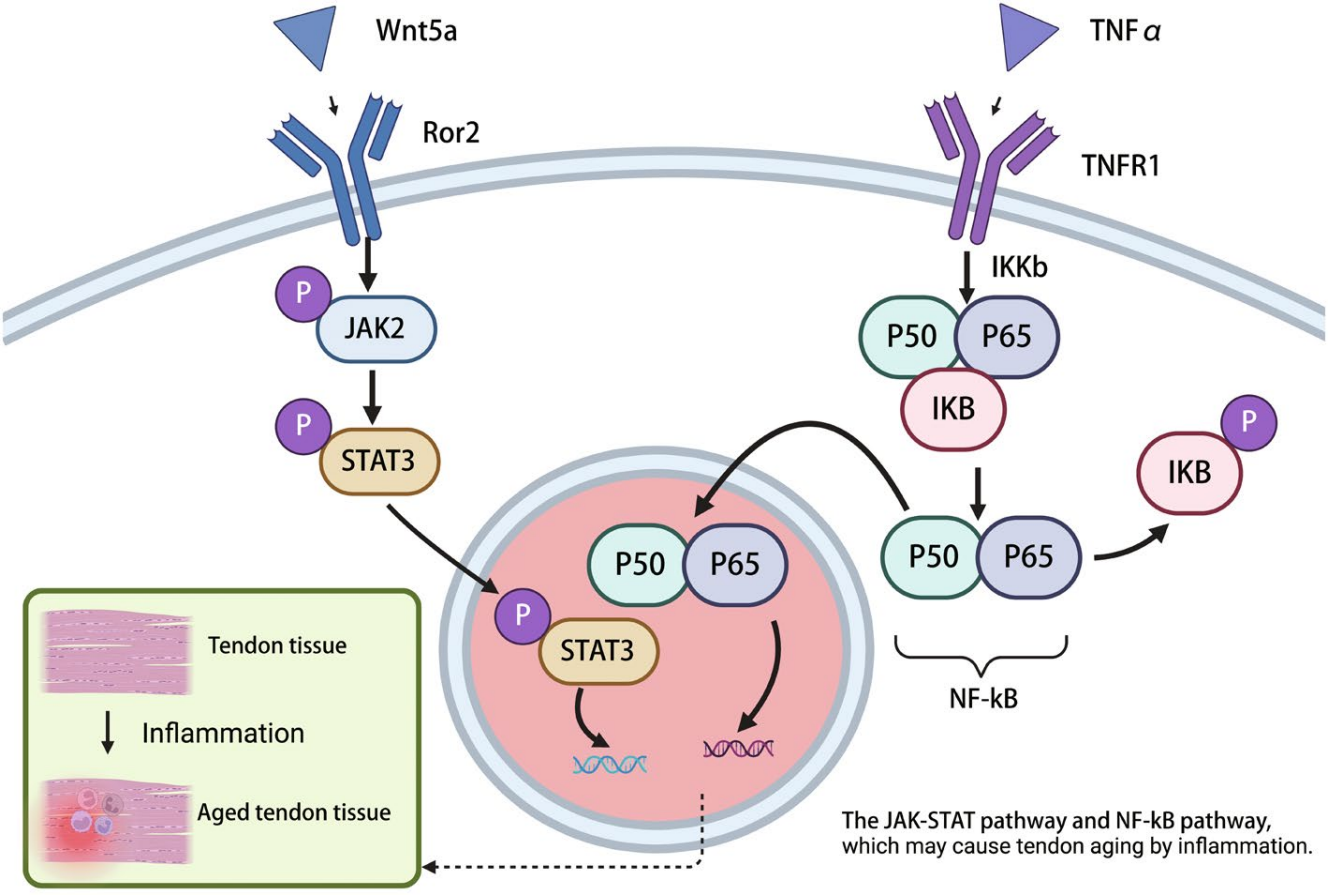
Describe inflammation‐related signaling pathways cause cellular aging, for example JAK–STAT signaling pathway and NF‐kB signaling pathway.

Aging‐related proinflammatory substances secreted by senescent cells contribute to the aging of TSPCs, impair their regenerative capacity, induce irreversible changes, and alter the bioactivity of surrounding cells. In women, age‐related tendon laxity increases the risk of accidents and inflammatory reactions [1]. In studies on Achilles tendon tendinopathy in mice, it was observed that aged tendons exhibited elevated expression of inflammation‐related biomarkers, including inflammatory disulfide HMGB1, macrophage marker CD68, and senescence‐associated markers such as SA‐β‐gal, p53, and p16 [63]. Based on these findings, it has been proposed that metformin may prevent HMGB1 translocation, reduce inflammation, and attenuate degeneration of aged tendons [63]. Furthermore, another study identified that the Ptges2 gene elevates the risk of tendinopathy by downregulating the expression of Cox2 [18] and promoting the production of prostaglandin H2 (PGE2), a signaling molecule implicated in tissue degeneration and inflammation. Thus, the inflammatory response occurring in tendons is believed to be driven by the over‐differentiation of adipocytes and the accumulation of preadipocytes and adipocyte progenitor cells in the tendons of aging individuals [33]. Moreover, aging cells release a range of molecular factors referred to as the senescence‐associated secretory phenotype (SASP), which includes chemokines and proinflammatory cytokines. SASP primarily exerts its effects on the surrounding tissue through paracrine signaling, thereby accelerating the aging process of adjacent cells and tissues [33]. Tendinopathy occurring with the SASP, the chronic injury or degeneration is typically associated with tendon thickening, instability, and an inflammatory response aimed at repair [64]. While a moderate inflammatory response following injury can aid tendon protection, persistent and excessive inflammation may impair tendon repair. During the early stages of inflammatory repair, TSPCs play a critical role in tendon recovery [65]. However, aged TSPCs may reduce the tendon's ability to differentiate and, in the presence of inflammatory responses, further contribute to degenerative tendinopathy [66], which could serve as a significant source of SASP [67]. In aged TSPCs, heightened activity of the JAK–STAT signaling pathway leads to age‐related dysfunctions in TSPCs renewal and migration [21]. The anti‐inflammatory cytokine interleukin (IL)‐10 is upregulated during the late inflammatory response. In vitro studies by Den [65] have shown that IL‐10 treatment enhances TSPCs proliferation and migration, but also activates the JAK/STAT3 pathway, which in turn inhibits the expression of tendon‐specific biomarkers. Therefore, future studies may seek to explore the beneficial effects of IL‐10 on TSPCs while mitigating its negative consequences. In contrast, the IL‐6 having proinflammatory effects; another study by Chen [68] reported that IL‐6 similarly activates the cell cycle and promotes the transition of TSPCs from the G1 phase to the G2/M phase, while inhibiting tenogenic differentiation. Ho's study [8] demonstrated that injecting a short peptide derived from pigment epithelium‐derived factor (PEDF) (PSP) activates the ERK2 and STAT3 signaling pathways, which are important in mediating inflammatory responses and promoting TSPC accumulation, thereby aiding tendon repair. Wnt5a modulates the JAK–STAT signaling pathway, which in turn facilitates the transition of the Wnt signaling pathway from the canonical to the noncanonical mode. The activation of the noncanonical Wnt pathway exacerbates the aging process in TSPCs. Cai [69] also found that celecoxib can slow TSPC aging by activating the canonical Wnt signaling pathway, with Ror2 functioning as a key receptor for Wnt5a in the aging of TSPCs and serving as an important mediator of Wnt5a‐induced aging [67]. Additionally, activation of the NF‐kB signaling pathway has been implicated in age‐related inflammation in degenerative tendinopathy [66]. NF‐kB is a critical regulator of the inflammatory initiation phase. Upon cellular stimulation by inflammatory stimuli, NF‐kB is activated, translocates to the nucleus, and binds to specific DNA sequences to initiate the transcription of inflammatory genes (Figure 3). RSPO2, specifically expressed in TSPCs, promotes tendon healing via the NF‐kB signaling pathway induced by either inflammation or mechanical loading, and inhibits heterotopic ossification, thereby maintaining tendon homeostasis [70]. Proinflammatory signaling pathways are commonly activated during aging, leading to inflammation. Novel approaches to combating tendon aging may involve targeting these pathways with specific inhibitors. Given the intertwined nature of inflammation and TSPC aging, future research should place particular emphasis on the NF‐kB and JAK–STAT signaling pathways. In brief, proinflammatory substances secreted by senescent cells contribute to the aging and impaired regeneration of TSPCs, accelerating tendon degeneration. Inflammation‐related signaling pathways play a central role in tendon aging and tendinopathy. These pathways not only promote tendon degradation but also exacerbate the SASP, which affects surrounding tissues. Therapeutically, targeting these inflammatory pathways using inhibitors such as metformin, celecoxib, or PEDF‐derived peptides may help slow TSPC aging and enhance tendon repair. Additionally, modulation of anti‐inflammatory cytokines like IL‐10 may offer dual benefits by promoting TSPC proliferation and migration while suppressing tenogenic differentiation. In conclusion, targeting key inflammatory pathways, particularly NF‐kB and JAK–STAT, holds promise as a therapeutic strategy for tendon aging. The inflammatory response in aging tendons promotes the excessive differentiation of mesenchymal cells into adipocytes, leading to fat infiltration within the tendon. Simultaneously, the elevated levels of inflammatory factors exacerbate oxidative stress and DNA damage, which ultimately activates apoptotic signaling pathways, accelerating cell death. This process disrupts tendon structure and accelerates aging, impairing tendon repair and regeneration.

### 3.4 Apoptosis

A study on age‐related cellular and microstructural changes at the rotator cuff attachment site revealed that the population of rotator cuff‐derived cells, including tendon cells, exhibits a reduced capacity for proliferation, damage repair, and tendon formation as they age [71]. Additionally, these cells show decreased migratory ability and reduced capacity to form cell clusters. According to Lee's studies [72], apoptosis can occur at any site within the rotator cuff. They found that apoptosis often coincides with tendon cell proliferation, suggesting that apoptosis may be linked to the process of tissue damage repair [73]. Palumbo's research [73] indicates that apoptosis requires substantial energy supply; however, tendon tissue, unlike muscle, has a limited blood supply to meet this energy demand, which could lead to degeneration due to natural changes. In tendons subjected to fatigue or injury, noninjured cells may undergo apoptosis due to cellular damage induced by mechanical loading; therefore, preventing apoptosis by forcing these cells to survive within a damaged cytoplasmic matrix may cause further harm. The natural aging process is accompanied by a reduction in tendon cell numbers and changes in their functional properties, which can hinder tendon healing and serve as a potential risk factor for chronic tendinopathy [34]. One key marker of apoptosis, P53, was found to be upregulated following rotator cuff tears [73]. Rho's study [74] found increased expression of apoptosis‐related proteins, such as Bax, Bcl‐2, caspase‐3, and caspase‐9, in tendon cells after injury. In Shinohara's study [55], imaging signal intensity ratios of the rotator cuff and deltoid muscle were analyzed, revealing significantly higher expression rates of oxidative and apoptotic cells in rotator cuffs with Type 3 tears compared to other tear types. Apoptosis is more likely to occur in hypoxic and other adverse environments [75], with excessive apoptosis identified as a major factor in the development of tendinopathy [76]. Zhang's research [77] demonstrated that exosomes secreted by TSPCs can be internalized by tendon cells to inhibit apoptosis and promote high‐quality tendon healing, suggesting that TSPCs play an important role in preventing apoptosis. However, significantly higher levels of apoptosis were observed in TSPCs within aged tendon tissues [78]. Aspirin, a nonsteroidal anti‐inflammatory drug (NSAID) commonly used to treat tendinopathy, has been shown to induce apoptosis in TSPCs through the Wnt/β‐catenin pathway by regulating mitochondrial and caspase‐3 function [79]. Elderly individuals with high cholesterol levels are also more prone to rotator cuff tears, as high cholesterol induces apoptosis in TSPCs via the ROS‐activated AKT/FOXO1 pathway [80]. Xu's study [60] found that Pio, a peroxisome proliferator‐activated receptor γ agonist, could induce apoptosis and compensate for this effect by activating autophagy in TSPCs, thereby improving TSPC dysfunction. Apoptosis in aged tissues may also affect TSPCs. Shi [81] found that rMFG‐E8 could enhance tendon healing by improving apoptosis regulation in tendon cells. In Yu's research [82], Ginsenoside RB1 was identified as a potential anti‐apoptotic agent, capable of delaying cell aging by regulating apoptosis. By combining anti‐apoptotic therapies, such as modulating mitochondrial function and activating autophagy, it is possible to delay cell senescence and promote tendon healing. Moreover, inflammation exacerbates the apoptotic process, suggesting that future strategies for treating tendon aging may focus on reducing apoptosis and improving cell function, particularly through the protection of TSPCs to enhance tendon repair and regeneration. Oxidative stress damages cells by generating reactive oxygen species (ROS), which trigger apoptosis and further accelerate tendon degeneration. In aged tendons, fatty infiltration is often closely associated with apoptosis, as excessive adipocyte differentiation and accumulation may be induced by oxidative stress, exacerbating cell death. Therefore, oxidative stress not only promotes apoptosis but also accelerates tendon aging through the induction of fatty infiltration.

### 3.5 Fatty Infiltration

With aging, senescent cells accumulate within adipose tissue [83]. The efficient production of mature adipocytes from mesenchymal progenitors is compromised, leading to the accumulation of immature adipocytes in surrounding tissue and contributing to fatty infiltration. This process is not only exacerbated by pain‐induced reduced activity but is also driven by the replacement of muscle tissue with adipose tissue due to tendon degeneration and protein degradation [84]. Fatty infiltration and fibrosis often accompany rotator cuff tears, with the extent of infiltration correlating with the rate of retear following surgical repair [85]. Therefore, the degree of fatty infiltration serves as an important prognostic marker for rotator cuff injury repair. Specifically, a Goutallier grade of 2 or higher is considered to significantly affect muscle function, and rotator cuff repair should be performed prior to reaching this stage, as it is a critical determinant of surgical outcomes [84]. Wilson [86] further established that the degree of fatty infiltration can act as a biomarker for chronic rotator cuff injuries, with elderly and female patients being more prone to this phenomenon. This excessive fatty infiltration is particularly prominent in muscle fibers and tissues following rotator cuff tears, although the precise molecular mechanisms remain unclear. Peroxisome proliferator‐activated receptor gamma (PPARγ) and CCAAT/enhancer‐binding protein alpha (C/EBPα) are closely linked to lipid metabolism and have been identified as potential biomarkers of adipogenesis following rotator cuff tears [87]. Fang [88] conducted bioinformatics analyses and experimental validation, revealing that Caveolin‐1 plays a pivotal role in the development of fatty infiltration after rotator cuff tears; additionally, overexpression of GATA6 was found to inhibit Caveolin‐1 expression and activate the cAMP/PKA pathway, thereby suppressing the adipogenic differentiation of TSPCs and reducing fatty infiltration. Recent studies have shown that TSPCs can differentiate into adipocytes through adipogenic differentiation, a process triggered by specific factors that induce adipocyte formation and subsequent fatty infiltration [89]. This phenomenon is particularly evident following tendon injuries and may be more pronounced in aging tendons, which exhibit a diminished capacity for repair compared to their younger counterparts. Although adipogenic differentiation in aged TSPCs has been shown to be heightened, inhibition of the PPARγ signaling pathway has been found to ameliorate this condition [90]. Fan's research further demonstrated that vascular endothelial growth factor (VEGF) could reduce adipogenic differentiation in TSPCs while enhancing angiogenesis in tendons, thus promoting healing in aged tendons [91]. In bioinformatics analyses, Hu [92] identified that the differential gene expression associated with fatty infiltration in the rotator cuff is linked to aging. As tendons age, their ability to form tendon and cartilage decreases, while their potential for adipogenesis increases [71]. Fibroadipose progenitor cells (FAPs) are primarily responsible for fatty infiltration but are also essential for muscle regeneration [93]. These cells are typically activated in response to muscle injury [94], and their numbers vary across different tissues [95]. FAPs tend to accumulate in areas with high vascularization and exhibit greater proliferative and adipogenic potential in normal rotator cuff tendons. Consequently, they are more prone to adipose infiltration following rotator cuff tears than tendons from other anatomical locations. Treatment with metformin after rotator cuff tears has been shown to activate inflammatory macrophages, inhibiting FAP differentiation or inducing FAP apoptosis, thus mitigating fatty infiltration [96]. Davies [85] demonstrated that apoptosis of FAPs could be promoted by inhibiting the TGF‐β signaling pathway, leading to reduced fatty infiltration in the rotator cuff. The fatty infiltration associated with aging is also attributed to hypoxic conditions following rotator cuff tears. Hypoxia‐inducible factors, HIF1α and HIF1β, enhance the promoter activity of fatty acid‐binding protein 4 (FABP4), thereby facilitating fat accumulation [97]. This suggests that fatty infiltration may be interconnected with metabolic diseases. Injection of vascular endothelial growth factor (VEGF) has been shown to reduce adipocyte accumulation in aged tendons, while targeting secreted phosphoprotein 1 (SPP‐1) can improve the biomechanical properties of tendons [91]. Thus, when addressing fatty infiltration, which contributes to tendon aging, it is crucial to consider not only the role of FAPs but also the aberrant differentiation of TSPCs. Suppressing the excessive adipogenic differentiation of TSPCs may help prevent fatty infiltration and mitigate further aging of the tendon tissue. Fatty infiltration is closely linked to tendon aging, particularly following rotator cuff injuries, and is associated with repair outcomes and retear risk. With aging, abnormal differentiation of TSPCs and FAPs promotes adipogenesis, impairing tendon repair. Interventions targeting adipogenesis pathways such as PPARγ inhibition or treatments like VEGF can reduce fatty infiltration and improve tendon healing (Tables 1 and 2).

**TABLE 1:** Studies related to mechanism of tendon aging.

Aging mechanism	Description	References
Oxidative stress	Oxidative stress has a passive effect on failed tendon healing of tendinopathies	[31]
	The rotator cuff tear tissues have more serious oxidative stress and tendon degeneration than no tear rotator cuff	[36]
	NOS activity was higher in injured tendon healing than normal tendon	[37]
Hyperglycemia induction	AGEs will disrupt tendon fibroblast homeostasis and develop the diabetic tendinopathy	[98]
	The tendon fibroblast caused by AGEs, and result in the diabetic tendinopathy	[53]
	In rotator, cuff tear tissues have a lot of AGEs accompany with the highest oxidative stress and apoptosis	[99]
	AGEs would diminish tendon collagen fiber sliding, also have the mechanical effect on a loss of tissue viscoelasticity, with aging	[56]
	Fiber sliding would be limited through tissue viscoelasticity decline by AGEs	[56]
	Proteoglycan deposition increases in old groups compared with young or adult groups	[14]
Inflammatory reaction	NF‐KB pathway activated will cause the age‐related inflammation to induce TSPCs aging, and rotator cuff deteriorate	[66]
	The JAK–STAT signaling pathway was activated in aged TSPCs	[21, 67]
Apoptosis	With aging, the rotator cuff‐derived cells' ability of proliferation, migration, and other functions will be weakened	[71]
	During normal aging, the number of tenocytes will reduce, and the properties will change	[34]
	Apoptosis was positively correlated with the severity of tendinopathy	[76]
Fatty infiltration	The number of FAPs and the fibrogenic differentiation are related to age and the tear size	[84]
	Age and female gender are risk factors for fatty infiltration in normal rotator cuffs	[86]
	The adipogenicity potential increased in rotator cuff‐derived cells with age	[71]
	FAPs activated by muscle injured and would interact with inflammatory	[94]

**TABLE 2:** Role of TSPCs in tendon aging.

TSPCs in tendon aging	References
Bone morphogenetic protein with aging promote the osteogenic differentiation ability of TSPCs	[40]
Oxidative stress would impair the viability of TSPCs, effect the tendon healing	[45]
Accumulation of AGEs would enhance ossification of TSPCs, result in heterotopic ossification in tendon	[60]
High glucose led to decreased TSPCs viability and promote osteogenic differentiation of TSPCs	[61, 62]
Inflammatory factors affect the ability of TSPCs to proliferate and migrate, and tendon formation	[65, 68]
The adipogenic differentiation of aged TSPCs was also found to be elevated	[90]

## 4 Therapeutic Modalities and Outlook for Tendon Aging

### 4.1 Promote Normal Differentiation of TSPCs

Tendon injury is more likely to occur in aged tendon, which is one of the reasons for postoperative retear and poor function. The primary barrier to the use of stem cells in the treatment of patients with tendinopathy is their ability to differentiate into tendon cells. Abby's [98] experiments revealed that tendon tensile loading and a linear fiber environment encourage this process [3]. Being a cell with diverse differentiation capacities, TSPCs are crucial to the aging of tendons, and their misdifferentiation would accelerate the aging process of tendons. Additionally, Yu's [100] work discovered that the TSPCs' adipogenic and osteogenic differentiation may be modulated by the lncRNA KCNQ1OT1/miR‐138/PPARγ or RUNX2 axis, so this axis is also a potential future therapeutic direction. In studies on diabetic calcific tendinopathy, it was observed that high‐oxygen environments induce calcification in rat Achilles tendons; blocking the ROS/HIF‐1α signaling axis was shown to inhibit the osteogenic differentiation of TSPCs [101]. The Hedgehog signaling pathway contributes to injury‐induced ectopic ossification in tendons and regulates osteogenesis through antioxidant pathways in tendon‐derived stem cells. Therefore, Hedgehog signaling is also a critical regulator of the aberrant differentiation of tendon stem cells [102]. As discussed earlier, abnormal tendon microenvironments contribute to the dysfunctional differentiation of TSPCs. However, the injection of healthy TSPCs can promote matrix synthesis and collagen production in tendons. Although direct injection does not produce optimal results, DNA hydrogel‐based delivery systems have been shown to enhance TSPC retention in the tendon microenvironment [103], leading to better outcomes than those achieved with free TSPCs. This approach also alleviates abnormal differentiation of TSPCs. Additionally, these delivery systems can be combined with therapeutic agents to further promote TSPC differentiation and delay tendon aging.

### 4.2 Resistance to Oxidative Stress and Control of Blood Glucose

Oxidative stress is a common consequence of tendon aging. Exercise therapy, when combined with antioxidant supplementation, may more effectively enhance the body's antioxidant capacity [44]. Future treatment strategies may involve the use of various antioxidants; however, current research has not identified the most beneficial type of antioxidant supplement. Vitamin C is a well‐known antioxidant. According to research, supplementation with collagen‐rich gelatin containing vitamin C prior to intermittent activity can promote collagen synthesis [104]. Platelet‐rich plasma (PRP), which is abundant in growth factors that promote tissue regeneration, is commonly used in tendon injury treatment. In addition to its regenerative effects, PRP also possesses antioxidative properties. It achieves this by activating the Nrf2 pathway, which facilitates tendon regeneration and repair [105]. Peroxiredoxin 5 (PRDX5), which exhibits differential expression between normal and degenerative tendons, is also considered a key molecular target for combating oxidative stress in tendon tissue [106]. In a study by Ren [107], cerium oxide nanoparticles carrying human umbilical cord mesenchymal stem cells were found to counteract oxidative damage and promote tendon regeneration. Furthermore, the administration of antioxidants can help reduce advanced glycation end‐products (AGEs) caused by oxidative stress. However, diabetic patients should still use medications such as metformin to control blood glucose and prevent diabetic tendinopathy [9]. The combined use of antidiabetic medications and antioxidants should be considered in treatment strategies for diabetic patients.

### 4.3 Control of the Inflammatory Response

The inflammatory response and apoptosis brought on by modifications in mitochondrial dynamics may be mitigated by mitochondrial transplantation [108]. One of the primary causes of tendon aging is the inflammatory response. Chen [67] showed that age‐related dysfunction in TSPCs could be restored by inhibiting the JAK–STAT signaling pathway through knockdown of JAK2 or STAT3 [21]. This inhibited the activation of the JAK–STAT signaling pathway by Wnt5a, which decreased the expression of SASPs in TSPCs. The activation of the JAK–STAT signaling pathway was suppressed by overexpressing aquaporin 1, indicating that aquaporin 1 improves aging and age‐associated dysfunction in TSPC by blocking the JAK–STAT signaling pathway [109]. Inhibition of the JAK–STAT pathway is a major pathway to ameliorate inflammatory responses, and the use of relevant anti‐inflammatory drugs may improve the age‐related inflammation of TSPCs, which could delay the degenerative tendinopathy, and the exosome derived from TSPCs could also adjust the inflammation to promote the tendon healing in high quality [77]. By downregulating PTEN/PI3K/AKT signaling, aspirin was utilized to manage inflammation that might suppress adipogenesis and fatty infiltration of TSPCs in injured tendons, promote biomechanical characteristics, and lower the risk of rupture in tendon injuries, all of which could be used to treat tendon aging. Tendons' mechanical qualities can be enhanced, their density and stiffness may be increased, and their ability to store and release elastic energy can be improved by collagen and vitamin C [110]. Another signaling pathway linked to inflammation is NF‐kB, and TSPC aging can be improved in vitro by inhibiting the IKKB/NF‐kB signaling pathway [66].

In the study by Chen [111], it was found that injectable self‐healing hydrogels with sustained release of Mg^2+^ and curcumin exhibited anti‐inflammatory properties and promoted tendon healing. In another study by Zhao [112], it was demonstrated that using tannic acid (TA) to modify decellularized tendon slices (DTS) to create functional scaffolds DTS‐TA with antioxidant and anti‐inflammatory properties effectively alleviated inflammation and promoted tendon regeneration. This was achieved by increasing the M2/M1 macrophage ratio and the expression of IL‐4, reducing the secretion of IL‐6 and IL‐1β, as well as clearing excessive ROS both in vitro and in vivo.

### 4.4 Improvement of Fatty Infiltration

High‐load training has been shown to preserve the mechanical properties of tendons without altering the collagen fibers within them [113]. Moreover, appropriate exercise can enhance muscle regeneration by promoting the aging of fibro‐adipogenic progenitors (FAPs) [114]. Aging tendons are often accompanied by fatty infiltration, and FAPs may accumulate excessively following tendon injury. Interleukin‐15 has been found to stimulate the proliferation of FAPs and inhibit their adipogenesis both in vitro and in vivo [93]. Furthermore, the injection of leukocyte‐poor platelet‐rich plasma (PRP) has been demonstrated to effectively reduce the retear rate and improve Goutallier grading in patients following arthroscopic rotator cuff repair [115]. Additionally, inhibition of the transforming growth factor β (TGF‐β) signaling pathway may induce apoptosis in FAPs, thereby reducing the FAP population and mitigating fibrosis and fatty infiltration in the rotator cuff [85]. Given its potential link to metabolic processes, the PI3K‐AKT and MAPK signaling pathways, both of which are associated with lipid metabolism, may represent important regulatory pathways in this context [116].

### 4.5 Other Potential Therapeutic Targets for TSPCs

The Rho‐associated coiled‐coil containing protein kinase (ROCK) is closely associated with cytoskeletal dynamics and cellular migration. Inhibition of ROCK has been shown to restore aged tendon stem/progenitor cells (TSPCs) to a more youthful phenotype, characterized by smaller cell size, restored actin dynamics, and enhanced migratory capacity [26]. Additionally, the overexpression of miRNA‐135a in TSPCs has been demonstrated to prevent cellular aging by promoting tendon‐specific differentiation, migration, and proliferation, so miRNA‐135a targets ROCK1 and plays a significant role in the aging process of TSPCs [117]. Rui's study [23] identified connective tissue growth factor as a potential therapeutic target for treating aging TSPCs. The injection of connective tissue growth factor has been found to significantly impact aging TSPCs and may serve as a promising molecular treatment for age‐related tendon disorders [23]. This treatment promotes tendon healing by stimulating the proliferation of TSPC populations [8]. Moreover, appropriate mechanical stimulation can modulate the disassembly and composition of the tendon extracellular matrix, providing a foundation for tendon metabolism and mechanical properties, this also promotes the expression of genes related to matrix metalloproteinases (MMPs) such as MMP9, MMP13, and MMP14 in TSPCs, which is mediated by the ERK1/2 and p38 signaling pathways within the MAPK pathway [4]. Furthermore, the use of biological materials for the targeted delivery of various antiaging treatments should be considered to improve the sustained release and efficacy of therapeutic agents. To minimize adverse reactions and enhance the therapeutic effects on tendon aging in a more focused manner, local administration should be recommended for tendon‐targeted treatments.

## 5 Summary

Aged tendons exhibit a diminished capacity for repair, primarily due to the intrinsic limitations of tendon tissue's self‐healing potential, which is constrained by its unique structural composition. Following injury, this often accelerates the cycle of tendon degeneration. The therapeutic effects of pharmacological interventions and exercise on tendon aging may be limited. Therefore, it is imperative to investigate the underlying mechanisms of tendon aging, including oxidative stress, hyperglycemia‐induced aging, inflammation, apoptosis, and fatty infiltration. Although several therapeutic strategies have been proposed for tendon aging, each offers distinct advantages. Promoting the normal differentiation of TSPCs while preventing their aberrant differentiation into adipocytes or osteoblast‐like cells can address cellular dysfunction at its origin. Compared with conventional anti‐inflammatory or antioxidant treatments, this approach is more effective in restoring the structure and function of tendons. Antioxidant therapies directly mitigate tendon damage induced by oxidative stress, with enhanced efficacy when combined with exercise therapy. This approach combats the free radical damage associated with aging, while simultaneously boosting the body's antioxidant defenses to promote tendon self‐repair. Controlling inflammation plays a critical role in modulating the microenvironment of aging tendons and can be integrated with other therapeutic strategies to suppress excessive immune responses, thereby facilitating tendon regeneration and repair. Therapeutic interventions targeting fatty infiltration, by regulating the proliferation and differentiation of FAPs, effectively reduce fat accumulation in aging tendons, thereby preserving their mechanical properties. This strategy directly addresses the fatty infiltration characteristic of tendon degeneration, making it particularly suitable for age‐related tendon pathologies. Each therapeutic strategy provides unique advantages in addressing tendon aging, and a targeted, condition‐specific treatment approach is essential. Such tailored strategies offer a more comprehensive defense against tendon aging, ultimately improving clinical outcomes. Understanding the underlying causes of tendon aging allows us to slow or potentially prevent its progression, thereby improving tendon structure and function. Aging tendons are more susceptible to injury and exhibit a reduced capacity for healing postinjury; however, tendon injury does not necessarily indicate that the tendon is aging. Tendon aging is associated with pathological changes at the cellular and tissue levels, which lead to a general decline in tendon structure and function. This article emphasizes the role of TSPCs in tendon aging, but it should not be construed as suggesting that research in this area should be limited. We hope that future studies on tendon aging will continue to explore the role of TSPCs and investigate the mechanisms underlying tendon aging to develop novel therapeutic strategies aimed at slowing the aging process and enhancing tendon function.

## Author Contributions

Yinxian Yu performed the study concept and design. Wenhui Gu and Guohua Wang performed the development of methodology and writing. Haifeng Zhang performed the review and the revision of the paper. All authors read and approved the final paper.

## Conflicts of Interest

The authors declare no conflicts of interest.
